# Patterns of cardiovascular variability after long-term sino-aortic denervation in unanesthetized adult rats

**DOI:** 10.1038/s41598-018-37970-0

**Published:** 2019-02-04

**Authors:** Alberto Radaelli, Giuseppe Mancia, Caterina De Carlini, Francesco Soriano, Paolo Castiglioni

**Affiliations:** 1 Cardiac Rehabilitation, S. Gerardo Hospital, Monza, Italy; 2 Università Milano Bicocca, Monza, Italy; 3 IRCCS Fondazione Don Carlo Gnocchi, Milan, Italy

## Abstract

Baroreflex dysfunction is a diffuse chronic condition that is expected to be followed by a profound loss of organization of BP and HR variability. Nevertheless, long-term effects of baroreflex withdrawal are still debated. Aim of our work was to study BP and HR changes long term after sino-aortic denervation (SAD). Inter-beat-interval (IBI) and intra-arterial BP were recorded beat-by-beat in 43 Wistar-Kyoto rats (Controls, n = 33; SAD rats, n = 10). Power spectra were calculated in controls and in SAD rats within three days and at seven months from denervation. Compared to controls, chronic SAD rats showed 1) similar mean BP (control vs SAD: 95 ± 16 vs 87 ± 22 mmHg) and IBI (171 ± 22 vs 181 ± 15 ms) values, 2) dramatically higher values of BP variance (12 ± 2 vs 64 ± 2 mmHg^2^, p < 0.01) and of ultra- (ULF) and very-low-frequency (VLF) BP oscillations, 3) dramatically higher values of IBI variability (24 ± 2 vs 71 ± 4 ms^2^, p < 0.01) and of ULF-IBI oscillations that were synchronized with BP oscillations. Chronic SAD rats reveal a marked change in the pattern of cardiovascular variability characterized by the appearance of synchronized slower oscillations of BP and HR. The cardiovascular system, therefore, retains a high level of organization despite the absence of a reflex control mechanism.

## Introduction

The baroreceptor reflex plays a crucial role in the preservation of cardiovascular homeostasis as demonstrated by the striking increase in blood pressure (BP) variability that follows sino-aortic denervation (SAD) in animals^1–4^. In absence of a functioning baroreflex, the cardiovascular patterns of BP and heart rate (HR) variability are therefore expected to change. As baroreflex dysfunction is a diffuse condition affecting ageing, deconditioning and different diseases (like hypertension, heart failure, diabetes mellitus, coronary disease, obesity and Parkinson’s disease)^5^ and as changes in cardiovascular patterns could have consequences on prognosis^6–9^, the chronic effects of baroreflex dysfunction on both BP and HR should be carefully investigated^5^. Nevertheless, it is still not clear whether and to what extent baroreflex withdrawal affects BP and HR in the long term. Most of the studies that reproduced baroreflex withdrawal by SAD in animals in fact have been characterized by one or more limitations, such as (1) the short observation period after SAD, (2) the failure to document the completeness and persistence of the baroreflex arch inactivation, (3) the limited amount of observations on the components of BP variability affected by baroreflex denervation and (4) the incomplete information on the effects of SAD on HR and HR variability particularly over the long-term after SAD^2,10–20^. The last question is not of minor importance because knowledge of both BP and HR effects of SAD may clarify the hemodynamic consequences of baroreceptor denervation at vascular versus cardiac level. Aim of our study was, therefore, to (1) extend the observations made after surgical SAD to a period longer than that of most previous studies; (2) determine the post-denervation changes in both BP and HR, and (3) avoid or minimize the problems and limitations of previous studies. For this aim, we analyzed beat-by-beat recordings of intra-arterial BP (MBP) and of inter-beat interval (IBI) both in control and in SAD rats one day, three days and seven month after denervation.

## Results

### Blood pressure, heart rate and respiratory rate

Three days after SAD, MBP was significantly higher in the denervated rats, but it subsequently showed a decrease and seven months after SAD its value was similar in the denervated and in the control animals; IBI was significantly shorter one day after SAD compared to control rats, but it subsequently increased and seven months after SAD it was similar to the value of the control animals (Table 1).

**Table 1:** MBP and IBI mean and variance and respiratory rate in control and SAD groups.

	Control	1-day SAD	3-day SAD	7-month SAD
**Mean**				
MBP (mmHg)	95.3 (16.3)	95.2 (15.7)	103.1 (17.3)*	86.7 (22.1)
IBI (ms)	171.1 (22.5)	144.8 (15.5)*	160.0 (25.0)	181.7 (15.8)
**Variance**				
MBP (mmHg^2^)	11.6 (1.8)	58.6 (2.0)**	61.3 (1.7)**	63.8 (1.7)**
IBI (ms^2^)	24.1 (1.9)	8.3 (1.9)**	17.4 (2.5)	71.0 (4.1)**
**Respiratory Rate**				
Spectral Estimate (breath/min)	96.0 (19.1)	82.5 (14.0)	102.7 (18.0)	98.9 (9.6)

Values as mean (SD) for MBP, IBI mean value and for respiratory rate, as geometric mean and geometric SD for variance. The * and ** indicate differences vs. controls significant at p < 5% and p < 1% respectively by unpaired t-test after log-transformation for variance, by Mann Whitney U test for the respiratory rate.

**Figure 1:**
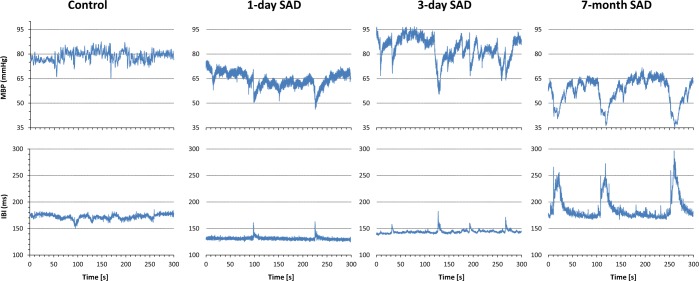
Five-minute segments of MBP and IBI series in a control rat and in a SAD rat at day 1, at day 3 and at 7 months from denervation. MBP variability, with blood pressure sudden reductions, clearly increases immediately after SAD and remains high thereafter. On the other hand, IBI variability, that decreases immediately after SAD, thereafter increases. The increase in IBI variability is sustained by sudden IBI prolongations that coincide with blood pressure reductions.

MBP variance was strikingly higher in SAD than in control rats at any time from SAD. In contrast IBI variance was significantly lower in SAD than in control rats at day 1 from SAD, but it thereafter showed a marked increase and at seven months from SAD it was significantly higher than in controls (Fig. 1 and Table 1).

The respiratory rate of SAD rats did not show significant differences from controls except for a non-significant reduction (p < 0.10) one day after SAD (Table 1).

### Spectral powers of blood pressure

**Figure 2:**
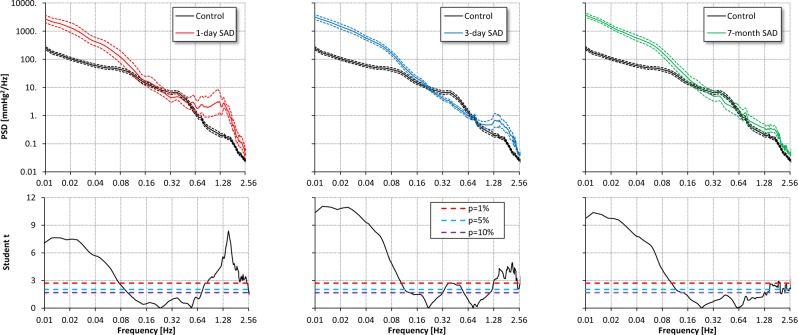
Comparison of MBP power spectra in the control group (black) vs. SAD groups 1 day (red), 3 days (light blue) and 7 months (green) after denervation. Upper panels: geometric mean ± geometric standard error, plotted in log-log scale; lower panels: Student *t* statistics for each spectral line, with 10%, 5% and 1% statistical thresholds (dashed horizontal lines). At frequencies where the Student *t* is above the threshold, the difference between groups is statistically significant. Spectral lines below 0.1 Hz have greater amplitude in SAD rats than in controls at any time from denervation; spectral lines above 1 Hz are greater in SAD rats, the difference being more pronounced in the acute than in the chronic phases. Interestingly, a spectral peak in control rats at 0.38 Hz (i.e., in the LF band) disappears in SAD rats.

The power of MBP oscillations in the ultra-low frequency (ULF) and in the very-low frequency (VLF) range was strikingly higher in SAD than in control rats at any time from SAD intervention. In contrast, even in presence of an overall marked increase in BP variability, the power of low-frequency (LF) MBP oscillations either did not differ or it was lower in SAD than in control rats. SAD was also associated with a significantly higher power of the high-frequency (HF) oscillations of MBP at day 1 and 3 although not at seven months from SAD (Fig. 2 and Table 2).

**Table 2:** MBP and IBI spectral powers and SBP-IBI coherency in control and SAD groups.

	Control	1-day SAD	3-day SAD	7-month SAD
**MBP (mmHg**^**2**^)				
ULF (0.01–0.05 Hz)	3.5 (1.9)	37.2 (2.1)**	43.6 (1.8)**	44.3 (1.6)**
VLF (0.05–0.25 Hz)	4.7 (2.0)	11.0 (2.7)*	14.2 (1.7)**	15.3 (2.1)**
LF (0.25–0.55 Hz)	1.7 (2.3)	1.3 (2.2)	1.0 (1.5)*	1.2 (3.1)
HF (0.60–2.1 Hz)	0.5 (2.3)	4.1 (3.8)**	1.1 (2.4)*	0.9 (2.3)
**IBI (ms** ^**2**^ **)**				
ULF (0.01–0.05 Hz)	13.1 (2.0)	3.6 (2.3)**	8.5 (2.5)	42.9 (4.6)**
VLF (0.05–0.25 Hz)	5.0 (2.2)	1.8 (2.2)**	4.7 (3.1)	10.5 (4.4)°
LF (0.25–0.55 Hz)	1.0 (2.2)	0.5 (2.1)°	1.0 (2.7)	2.2 (4.4)*
HF (0.60–2.1 Hz)	2.8 (1.8)	1.4 (1.5)*	1.6 (2.2)*	6.8 (2.8)**
**SBP-IBI coherency modulus**				
ULF (0.01–0.05 Hz)	0.52 (0.25)	0.63 (0.25)	0.57 (0.29)	0.59 (0.26)
VLF (0.05–0.25 Hz)	0.56 (0.19)	0.43 (0.23)	0.45 (0.17)	0.30 (0.15)**
LF (0.25–0.55 Hz)	0.46 (0.16)	0.34 (0.18)	0.21 (0.07)**	0.19 (0.10)**
HF (0.60–2.1 Hz)	0.37 (0.16)	0.54 (0.13)*	0.31 (0.12)	0.28 (0.12)
**SBP-IBI coherency phase (rad)**				
ULF (0.01–0.05 Hz)	−1.87 (0.96)	−2.97 (0.87)	−2.68 (0.89)	−2.49 (0.48)*
VLF (0.05–0.25 Hz)	−1.08 (0.59)	−2.60 (1.20)*	−2.63 (0.90)*	—
LF (0.25–0.55 Hz)	−0.59 (0.54)	−0.62 (1.61)	—	—
HF (0.60–2.1 Hz)	0.17 (1.27)	2.98 (1.26)**	—	—

Spectral powers as geometric mean and geometric SD; coherency phase as circular mean and circular SD; coherency modulus after Fisher’s z transform; the °, * and ** indicate differences vs. controls significant at p < 10%, p < 5% and p < 1% respectively by unpaired *t*-test after log-transformation of power spectra. Coherency phases estimated only when the coherency modulus is greater than C_th_, and compared by Mann Whitney U test.

### Spectral powers of heart rate

**Figure 3:**
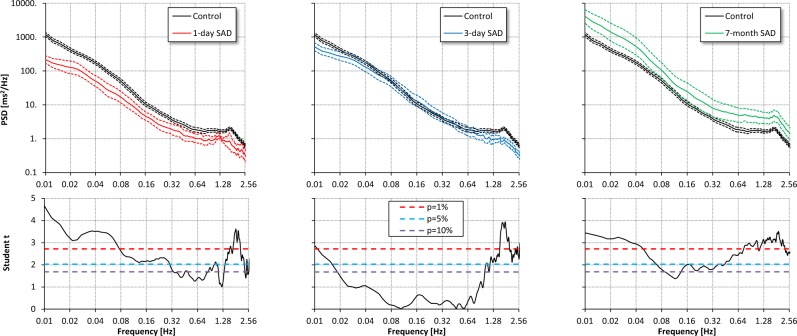
Comparison of IBI power spectra in the control group (black) vs. SAD groups 1 day (red), 3 days (light blue) and 7 months (green) after denervation. Upper panels: geometric mean ± geometric standard error, in log-log scale; lower panels: Student *t* statistics (see also Fig. 2). Compared to controls, the spectrum is significantly lower one day after SAD. Thereafter the power of the spectrum of SAD rats increases to become significantly higher 7 months after SAD almost at all the frequencies.

The powers of the ULF and VLF oscillations of IBI were significantly lower in SAD than in control rats at day 1 from SAD, but thereafter ULF oscillations showed an increase and became significantly higher than in controls seven months after SAD. The power of LF IBI oscillations followed a similar pattern, and this was the case also for the power of the HF IBI oscillations (Fig. 3 and Table 2).

### Blood pressure – heart rate coupling and baroreflex sensitivity

**Figure 4:**
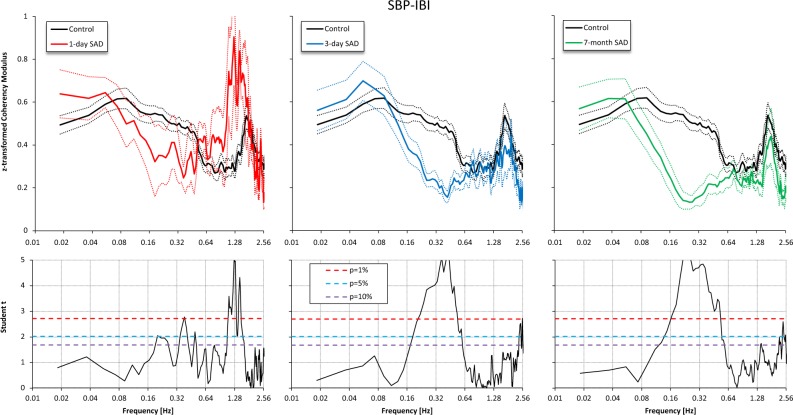
Coherency Modulus after Fisher z-transform in the control group (black) vs. SAD groups 1 day (red), 3 days (light blue) and 7 months (green) after denervation. Upper panels: mean ± standard error. Lower panels: Student *t* statistics (see also Fig. 2). The coherency modulus in the LF band is lower after SAD, the difference with controls being highly significant 3 days and 7 months after SAD; by contrast, the coherency modulus in the ULF band is high in all SAD groups, similarly to controls.

The coherency modulus between systolic BP (SBP) and IBI was significantly lower in SAD than in control rats in the LF range, particularly at day 3 and at 7 months from SAD, while it was similar to that of controls in the ULF range, whatever the time considered from SAD (Fig. 4 and Table 2).

**Figure 5:**
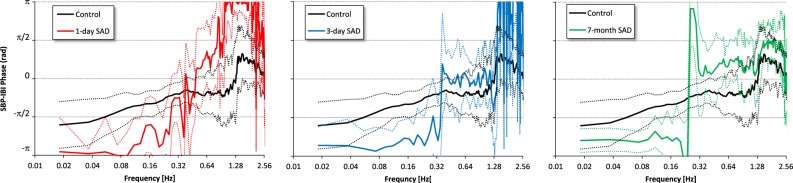
SBP-IBI phase in the control group (black) and in SAD groups 1 day (red), 3 days (light blue) and 7 months (green) after denervation. Circular mean and ±circular standard error: note the high uncertainty of estimates at frequencies where the coherency modulus is low (Fig. 4); in the ULF band, where it is high, the phase is close to −π/2 [rad] in controls, and to −π [rad] in all SAD groups.

The phase in the ULF and VLF ranges was lower in SAD than in control rats, being it close to −π rad in SAD rats and close to −π/2 rad in control rats at all the times from denervation (Fig. 5 and Table 2). The difference was statistically significant in the VLF band at day 1 and 3 from SAD, and in the ULF band at seven months from SAD. The phase close to −π in SAD rats means that the maximum in the SBP oscillation corresponds to a minimum in the IBI oscillation with the same frequency and a minimum in SBP occurs simultaneously to a maximum in IBI. Therefore a maximum in SBP value corresponds to a maximum in heart rate and *vice versa*. A different phase from controls also characterizes the respiratory oscillation 1 day after SAD.

The transfer function technique was unable to provide a measure of baroreflex sensitivity (BRS) in two control rats only (6% of cases). In the remaining 31 control rats, BRS mean value (SD) was 2.29 (0.82) ms/mmHg. By contrast, the percentage of rats in which the transfer function method was unable to provide an estimate raised to 80% one day and three days after denervation (p ≤ 0.001 vs. controls by Fisher’s exact test), and to 100% (p ≤ 0.001) seven months after denervation.

### Effects of stress after SAD

**Figure 6:**
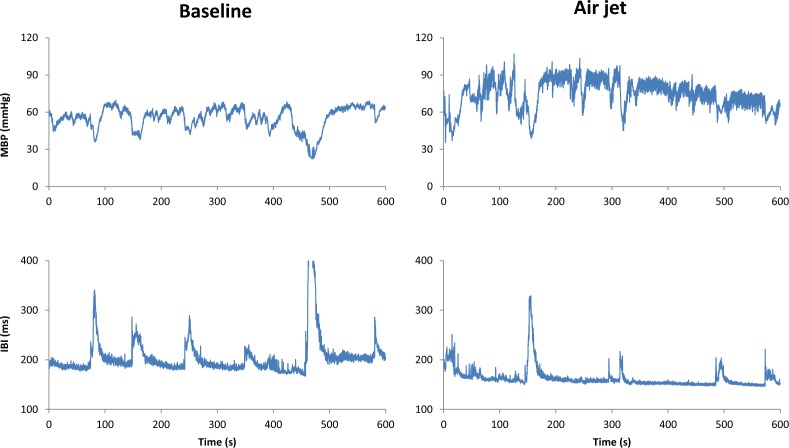
Ten-minute segments of MBP and IBI series in a SAD rat 7 months after denervation, in baseline and during a stressful stimulus. Note that even if the stressful stimulus may modify the general behaviour of the rat, the pattern of ULF variability during stress is similar to that of the baseline condition, i.e., with MBP sudden reductions and IBI prolongations that coincide with blood pressure reductions. In addition, the stressful stimulus increases the MBP level and the amplitude of MBP faster LF oscillations.

In SAD rats the air jet induced a significant increase in MBP as well as in the power of the LF oscillations of MBP both at day 3 and at seven month from SAD (Table 3), with no concomitant effect on ULF and VLF oscillations of MBP throughout. The air jet also did not significantly change the IBI mean level or powers (Table 3 and Fig. 6).

**Table 3:** MBP and IBI means and spectral powers in baseline and stress condition for each group of SAD rats.

	1-day SAD		3-day SAD		7-month SAD		Factors p		
	*Baseline*	*Air jet*	*Baseline*	*Air jet*	*Baseline*	*Air jet*	*Time*	*Stress*	*Interaction*
**MBP**									
mean (mmHg)	95.2 (15.7)	105.5 (21.5)	103.1 (17.3)	129 (23.8)**	86.7 (22.1)	122.2 (30.5)**	0.35	<0.01	<0.05
variance (mmHg^2^)	58.6 (2)	57.4 (2.6)	61.3 (1.7)	53.6 (2.2)	63.8 (1.7)	65.3 (1.4)	0.88	0.81	0.92
ULF (mmHg^2^)	37.2 (2.1)	41.6 (2.7)	43.6 (1.8)	33.2 (2.6)	44.3 (1.6)	34.1 (1.4)	0.99	0.50	0.73
VLF (mmHg^2^)	11.0 (2.7)	11.5 (2.7)	14.2 (1.7)	14.3 (2.1)	15.3 (2.1)	24.3 (1.5)	0.30	0.38	0.54
LF (mmHg^2^)	1.3 (2.2)	1.0 (2.6)	1.0 (1.5)	1.8 (1.6)*	1.2 (3.1)	3.1 (1.8)**	0.27	<0.05	0.07
HF (mmHg^2^)	4.1 (3.8)	0.9 (10)*	1.1 (2.4)	1.4 (2.3)	0.9 (2.3)	1.7 (2.6)	0.71	0.42	<0.05
**IBI**									
mean (ms)	144.8 (15.5)	150.4 (17.3)	160.0 (25.0)	163.5 (22.2)	181.7 (15.8)	170.4 (15.7)	<0.05	0.86	0.21
variance (ms^2^)	8.3 (1.9)	12.5 (3.0)	17.4 (2.5)	17.2 (1.8)	71.0 (4.1)	49.7 (3.0)	<0.01	0.95	0.48
ULF (ms^2^)	3.6 (2.3)	5.8 (2.7)	8.5 (2.5)	7.5 (2)	42.9 (4.6)	23.4 (3.1)	<0.01	0.73	0.26
VLF (ms^2^)	1.8 (2.2)	2.7 (3.0)	4.7 (3.1)	4.2 (2.2)	10.5 (4.4)	7.5 (3.0)	0.06	0.97	0.47
LF (ms^2^)	0.5 (2.1)	0.9 (4.4)	1.0 (2.7)	1.0 (2.7)	2.2 (4.4)	1.9 (4.1)	0.16	0.68	0.62
HF (ms^2^)	1.4 (1.5)	0.8 (17.2)	1.6 (2.2)	2.1 (2.5)	6.8 (2.8)	7.4 (4.1)	<0.05	0.81	0.61

Powers and variance as geometric mean and geometric SD; the * and ** indicate differences between baseline and air-jet stimulus significant at p < 5% and p < 1%.

## Discussion

The main results of our study are that compared to control animals, chronic SAD rats showed (1) similar mean values of blood pressure and heart rate, (2) a dramatic increase in the overall variance in blood pressure with a huge increase in the power of ULF and VLF MBP oscillations and unchanged power of LF MBP oscillations, and (3) an unexpected dramatic increase in heart rate variability mainly due, as for blood pressure, to an increase in the ULF power of IBI oscillations.

### Blood pressure level

In our rats, MBP showed a significant increase shortly following SAD, with however, a subsequent return to a level that was superimposable to that of control rats for the remaining duration of the study. This is in line with the results of previous studies in different animal species which have also shown the BP level to remain substantially unmodified by the elimination of the inhibitory sympathetic influences of the baroreflex^2,13–20^. It should be emphasized that, compared to most previous studies, potentially confounding factors were avoided or better controlled. One, as we measured baroreflex sensitivity throughout the study duration and found it to be undetectable 7 months after denervation, we would exclude an incomplete baroreceptor denervation. Two, because SAD rats responded to a stressful stimulus (air jet) with a marked MBP increase, the possibility that after SAD a BP rise was prevented by damage to the sympathetic efferent system during the aortic denervation procedure^21^ is unlikely. Three, it is also unlikely that the duration of the study was too short to see a BP increase because: (a) an observation period of seven months after the denervation is among the longest available in SAD studies; and (b) the sequential BP changes after SAD were in a depressor rather than pressor direction, with little difference in the BP value between control rats and rats seven months after SAD. Finally, we measured BP intra-arterially, thereby collecting all values and avoiding the risk of missing periods during which BP might have been higher than the one reported. This is a criticism advanced for indirect BP measures via the tail cuff method, which has also the inconvenience that during the measurements rats’ restraint and exposure to higher temperatures raise BP considerably, possibly obscuring the effect of SAD on BP^22^. Thus, the most likely conclusion is that withdrawal of the inhibitory baroreceptor input on the sympathetic nervous system leads to only a transient BP rise which vanes and entirely disappears with time, the implication being that indeed this reflex mechanism is not involved in the long-term modulation of BP levels. We can speculate that this is due to an adaptation of the centers receiving the baroreceptor afferent fibers^18,23,24^ or that, in absence of the baroreceptors, a tonic inhibitory afferent drive to the vasomotor center is taken up by cardiopulmonary receptors with afferent fibers travelling in the vagus^25,26^. However, studies in cats have been unable to show an increase in BP after vagotomy was added to SAD^15^, which speaks against this possibility. It is also possible that after SAD central or other mechanisms start contributing to long-term BP control, and indeed a baroreceptor-independent regulation of BP in the ventrolateral medulla has been proposed^24,27^.

### Blood pressure variability

Confirming previous studies^2,3^, in our rats SAD was accompanied by a striking increase of BP variability which appeared shortly after the denervation procedure and lasted unchanged throughout the study duration. The BP buffering action of the baroreceptors as well as their major role in favoring BP stability are thus confirmed. Our study, however, adds information on the components of BP variability that are modified by SAD and thus on their major or minor modulation by the baroreceptors.

As shown previously^3,28–30^, in our rats SAD was associated with a huge increase in the ULF and VLF oscillations that are believed to depend on hormonal influences^31,32^, modulation of vasomotor tone^30,32,33^, body movements^19,34^, and deep breathing^35^. In contrast, SAD had a less constant and clear effect on the LF oscillations of BP which showed at various times after SAD either no change or a decrease. Because these oscillations are regarded to reflect baroreceptor modulation of sympathetic tone^28,36,37^, this may provide further support to the hypothesis that sympathetic activity was not chronically increased after SAD and that this accounts for the absence of a MBP rise. Interestingly, even if after SAD an LF peak was absent in the MBP power spectrum, nevertheless during the jet stimulus the power in the LF band increased significantly, as previously observed in rats 2 weeks after SAD^38^. This implies that such a pattern of sympathetic activation can be generated from sources other than the baroreflex, one of them being probably the central nervous system^39^.

### Heart rate and heart-rate variability

Available studies have devoted limited attention to the effects of SAD on mean HR values and HR variability, usually quantified as an overall value with no description of its spectral components. Furthermore, observations have been restricted to a relatively short time after the SAD procedure, with no possibility to see whether the BP recovery from the acute post-SAD hypertension is paralleled by HR changes. Our study provides new observations that fill some of these gaps. One, HR increased shortly after SAD, but then returned to a value similar to that of intact rats, in parallel with the behavior of BP. Two, as previously reported^28,36^, overall IBI variability showed a reduction shortly after SAD but then increased to a value that at the seven month was significantly greater than that of control rats. Three, the final increase was accompanied by, and largely accounted for, an increase in ULF oscillations of IBI that showed high coherence with SBP. This is an intriguing finding because baroreflex impairment has been associated with a reduced HR variability although this has been usually reported in short term SAD studies. That HR variability increases after SAD when the observation period is prolonged is a new finding. The clarification of these mechanisms will have to wait for a better understanding of the neural and not neural mechanisms controlling HR variations, and in particular of the nature and factors governing the ULF and VLF oscillations. At present, ULF and VLF oscillations of HR are believed to depend on thermoregulation^40^, deep breathing^27^, physical activity^41^ as well as on the intrinsic sinus node activity^42–46^. There have been reports, for example, of the ability of sino-atrial cells to exhibit a regular discharge pattern after denervation^47,48^. Moreover an intrinsic cardiac nervous system has been described^49,50^, and right atrial cells have been shown to be able to generate spontaneous activity unrelated to the cardiac cycle but sensitive to arterial BP changes^49,51^. The latter finding may explain the parallelism after SAD of the slower fluctuations of HR and BP. Whatever the mechanism involved, synchronization of HR and BP fluctuations in the ULF band could underlie important physiological implications^52,53^.

### Limitations and Conclusions

One could object that we compared rats aged one or two months (controls) with rats aged seven to eight months (chronic SAD). However, evidence is available that mean BP values and BP variability do not substantially change with aging^22^. Moreover, aged rats show a decreased and not increased heart rate variability^54,55^. Therefore we could have under- and not over-estimated the increase in heart rate variability.

We did not measure the activity of the rats in the cage. Nevertheless it is unlikely that movements could have interfered with the estimation of the power in the ULF ranges as (1) ULF oscillations are characterized by a decrease in both HR and BP (Fig. 1) while movements in SAD animals have been shown to induce a decrease in BP but not in HR^19,34^, (2) the pattern of ULF BP oscillations was the same one day, three days and seven months after denervation, (3) the increase in HR variability took time to occur after denervation, and (4) the described change in activity of the rats in presence of the stressful air-jet stimulus^56^ should have produced a drastic change in ULF oscillations, which instead was not observed (Table 2, Fig. 6).

As far as the analysis of data is concerned, it is possible that in the future our understanding of the mechanisms involved in cardiovascular regulation might benefit from the analysis of longer recordings^57,58^ and from time series analysis based on the quantification of complexity, that have been recently shown to provide promising results in acute SAD rats^59^.

In conclusion, we have shown that in absence of a reflex control mechanism adult rats show a profound change in the pattern of cardiovascular variability that is largely accounted for by the presence of slow frequency oscillations of BP and HR. As in the long term these oscillations synchronize and stabilize, they do not seem to represent simply the effect of the withdrawal of a control mechanism but instead the effect of an intrinsic ability of the cardiovascular system to reach a new type of organization.

## Methods

### Animals, surgery and ethical approval

All procedures conformed with Italian Government regulations on protection of animals used for scientific purposes; as requested for experiments performed in rodents before 2014, that is the case of our study, the Italian Ministry of Health was informed on the purpose and protocol of the experiments performed (Gazzetta Ufficiale, 18 February 1992, n.116 art 7). The procuration of animals, the husbandry and the experiments conform to the ‘European Convention for the Protection of Vertebrate Animals used for Experimental and other Scientific Purposes’ (Council of Europe No 123, Strasbourg 1985). We studied 43 Wistar Kyoto rats (Charles River Italia, Calco, Italy) aged 11-12-week to eight months. Rats were subdivided into two groups: control rats (n = 33) and rats subjected to SAD (n = 10). Animals were housed under a standard artificial 12 h light-dark cycle with water and food provided *ad libitum*. Rats were anaesthetized with ketamine (75 mg. kg^−1^) administered IM. The adequacy of the anaesthesia was judged by the lack of reflex in response to a firm tail pinch. Additional anaesthetic was administered during the surgery if necessary (25% of the original dose IM). All surgical procedures were conducted under aseptic conditions. In each rat polyethylene catheters were implanted in the femoral artery for BP recording and in a femoral vein for drug injections through a small skin incision. The catheters were tunnelled subcutaneously, exteriorized at the dorsal neck region, and kept patent by flushing with appropriate heparin solution (0.01% vol/vol). Following surgery, ampicillin (125 mg.Kg^−1^, IP) was administered. After surgery more than 24 hours were allowed for the animal to recover and acclimate to the experimental environment, consisting of a wide cage in which the rat could walk, explore, eat and drink *ad libitum*.

### Sino-aortic baroreceptors denervation

The neck was aseptically incised along the midline. The internal and external carotid arteries were freed from their connections with the surrounding structures and the carotid sinus nerves were cut by severing all tissues between their enclosure in a double ligature. The aortic nerves were separated from the vagi near the nodose ganglion and cut. Additional aortic fibers possibly travelling outside the aortic nerves were cut by isolating and stripping the common carotid arteries over the entire neck and by sectioning the cervical sympathetic trunks near the nodose ganglion. After the vessels were stripped they were painted with 10% phenol in ethanol. Once the procedure was completed bilaterally the incision was sutured and closed^12^. The effectiveness of the denervation was verified one day after surgery by observing the lack of a reflex bradycardic response to a pressor stimulus produced by a 2–4 μg/kg bolus intravenous injections of phenylephrine.

Thereafter, the persistence of baroreflex denervation at different times from SAD was not verified with the phenylephrine method but with the analysis of spontaneous fluctuations of BP and HR (transfer function technique, see below), as the phenylephrine method has numerous disadvantages in SAD rats. Phenylephrine, in fact, may (1) lead to stimulation of stretch-dependent cardiopulmonary receptors^60^ with inhibitory sympathetic effects similar to those elicited by the arterial baroreceptors^26^; (2) modify venous return and thus the preload to the heart^61^; (3) alter cardiac contractility^62^; (4) stimulate baroreceptor pathways regardless of the increase in arterial pressure^63^; and (5) have chronotropic effects^64^. These limitations may be even more important in SAD rats due to changes in beta and muscarinic receptors expression that occur with time from denervation^65,66^.

### Experimental protocol

Data were collected during daytime in an unanesthetized condition. The arterial catheter was connected to a pressure transducer (model P23 Dc, Gould- Statham, Oxnard, CA). The BP signal was displayed on a chart recorder (7D polygraph, Grass Instruments, Quincy, MA) and tape recorded for off line analysis. HR was visualized from the pulsatile pressor signal via tachographic beat to beat conversion.

The intra-arterial BP signal was continuously recorded in the baseline condition for at least 10 minutes in all rats. In the group of ten rats subjected to SAD, recordings were made three days after denervation (SAD 3d, n = 10). In most of them, it was possible to repeat the recordings seven months after denervation (SAD 7 m, n = 7). Furthermore, in half of the ten rats subjected to SAD, recordings were also performed one day after denervation (SAD 1d, n = 5). To evaluate the preservation of a sympatho-mediated response^38^, each baseline BP recording in SAD rats was followed by a BP recording of 10 minutes during delivery of a continuous air-jet stimulus. At the end of the study, animals were deeply anaesthetized and then air was injected transcardially.

### Data Analysis

BP was digitized at 12 bits with a sampling rate of 1000 Hz. SBP, diastolic BP and MBP were identified beat by beat from the digitized signal, MBP being the variable used to express the results. IBI at a given beat *n* was calculated as the distance between the time of occurrence of SBP of beat *n* and SBP of the following beat, *n* + 1^67^. The time series were visually inspected for removing premature beats and artefacts, and for selecting a data-segment for the following analyses where the cardiovascular system appeared in a steady-state condition, i.e., with homogeneous characteristics of the signal dynamics over time, in particular excluding evident changes in the underlying BP and heart rate levels.

Beat-to-beat data were resampled at 100 Hz for frequency-domain analyses. The Welch periodogram was calculated over 180-s long, linearly detrended, 90%-overlapped Hann running windows. Spectral lines were estimated between 0.006 and 3 Hz after broad-band smoothing^3^. The cross-spectrum between SBP and IBI was calculated similarly splitting the recording into 180-s long, linearly detrended, 90%-overlapped Hann running windows, and the coherency function, γ(*f* ), was estimated as ratio between cross-spectrum and the square root of the product of SBP and IBI spectra^68^. The magnitude-squared coherency function |γ(*f* )|^2^, is the SBP-IBI coherence. Since the phase of the SBP-IBI cross-spectrum, in radiants, is defined as multiples of 2π (a turn angle), phases were scaled between -π and +π. A negative phase indicates that SBP variations precede IBI variations, while a positive phase indicates that SBP variations follow IBI variations^69^. Power spectra were decomposed in four frequency bands: the ULF band, between 0.01 and 0.05 Hz; the VLF band, between 0.05 and 0.25 Hz; the LF band, between 0.25 and 0.55 Hz; and the HF band, between 0.60 and 2.1 Hz. Because the literature does not define univocally the LF band in rats^70–74^, we set its upper limit at 0.55 Hz on the base of the shape of the coherency modulus between SBP and IBI as estimated in our control group. The variance of the time series was calculated as integral of the power spectrum. The respiratory rate was estimated as the frequency of the highest peak of the SBP spectrum within the HF band.

BRS was evaluated to verify the persistence of baroreflex denervation at different times from SAD. We employed the transfer function technique, based on the hypothesis that a resonance frequency in the baroreflex due to the time delay in the response of the vascular smooth muscles produces a BP oscillation in the LF band^75^, and that this BP oscillation is followed by a HR oscillation with the same frequency^28^. Accordingly, BRS was estimated as the ratio between the SBP-IBI cross-spectrum modulus and the root-squared SBP spectrum in the LF band^76^, and expressed in ms/mmHg, provided that the SBP and IBI spectral components were coupled and that the SBP oscillations preceded the IBI oscillations. The first condition is met if the SBP-IBI coherency modulus is greater than a minimum threshold, C_th_. We fixed C_th_ at a value of 0.33 (i.e., squared coherency modulus = 0.11) on the basis of the analysis performed with real and surrogate data described in the statistical analysis paragraph 4.5. The second condition is met if the SBP-IBI phase is negative^77^. The phase was calculated only for spectral components with coherency modulus |γ(*f* )| greater than C_th_ and averaged over the LF band. The transfer function modulus was averaged over the LF band considering only components with |γ(*f* )| greater than C_th_ and with negative phase. If no such components were found in the LF band, or if the average LF phase was not negative, the BRS was considered to be indeterminate, otherwise it was estimated as the averaged transfer function modulus.

### Statistical Analysis

Baseline differences between control rats and each group of SAD rats were tested by unpaired t-test for mean values and spectral powers, after log-transformation of spectral powers to obtain symmetric distributions close to Normal^78^, and by the Mann Whitney U test for BRS and the respiratory rate. The Fisher’s z-transformation was applied to the coherency modulus, |γ(*f* )|, before calculating means and standard errors over the groups and before applying unpaired t-test comparisons, as suggested in^79^. The Fisher’s z-transformation calculates the inverse hyperbolic tangent of |γ(*f* )|:

$$\begin{array}{ccc}{\rm{z}} & = & {\rm{arctanh}}\,(|{\rm{\gamma }}({\rm{f}}\,)|)\\  & = & \frac{1}{2}\,\mathrm{ln}(\frac{1+|{\rm{\gamma }}({\rm{f}}\,)|}{1-|{\rm{\gamma }}({\rm{f}}\,)|})\end{array}$$

which results approximately normal^80^.

**Figure 7:**
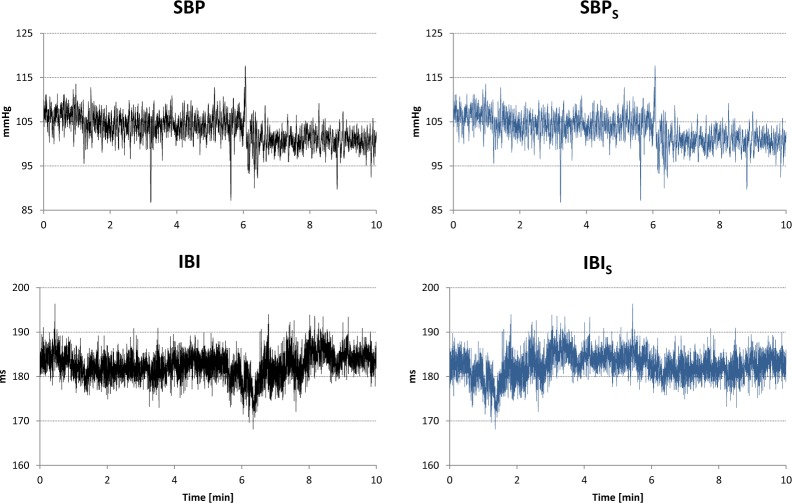
Generation of a couple of surrogate SBP_S_-IBI_S_ series from original SBP-IBI series. Left panels: example of *T* = 10 minute segment of SBP and IBI time series recorded in a control rats; right panels: corresponding surrogate series, obtained by exchanging first and second half of the IBI series.

**Figure 8:**
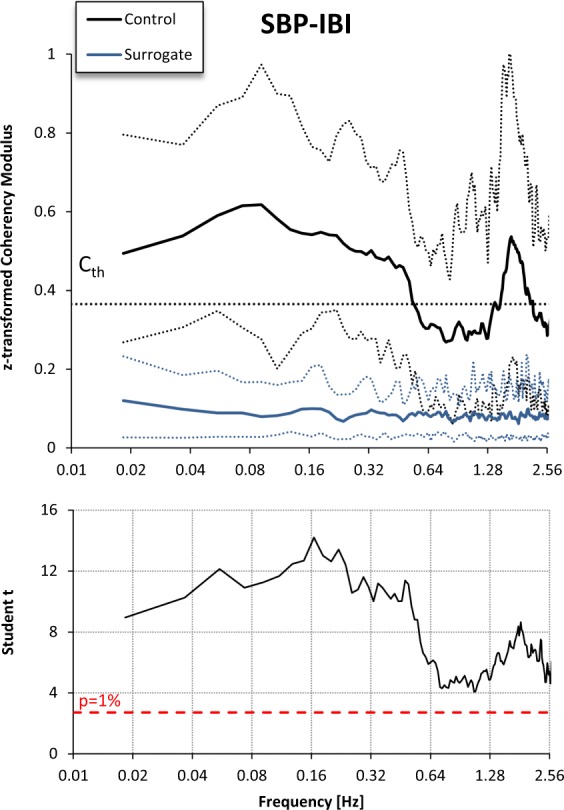
SBP-IBI Coherency Modulus after Fisher z-transform in the control group (black) vs. the corresponding surrogate series (blue). Upper panels: mean and tolerance limits as range between 10th and 90th percentile; the dotted horizontal line indicates the Cth threshold. Lower panels: Student t statistics for the paired comparison.

The statistical properties of coherency estimators were evaluated by making use surrogate data^81,82^. Following this approach, we generated a couple of surrogate series, SBP_S_(*t*) and IBI_S_(*t*) from each couple of SBP(*t*) and IBI(*t*) time series recorded in control rats as:

$$\begin{array}{ccc}{{\rm{SBP}}}_{{\rm{S}}}(t) & = & \mathrm{SBP}(t)\,{\rm{for}}\,{\rm{0}}\le t\le T\\ {{\rm{IBI}}}_{{\rm{S}}}(t) & = & \mathrm{IBI}(t+T/2)\,{\rm{for}}\,{\rm{0}}\le t < T/2\\ {{\rm{IBI}}}_{{\rm{S}}}(t) & = & \mathrm{IBI}(t-T/2)\,{\rm{for}}\,T/2\le t\le T\end{array}$$

with *T* duration of the series (see an example in Fig. 7). Surrogate series have the same probability density function and the same modulus of the discrete Fourier Transform of the original series, but they lack the SBP-IBI phase coupling because we exchanged first and second half of the IBI series. Figure 8 plots the coherency modulus |γ(*f* )|, calculated as described in paragraph 4.4, after z-transformation for both original and surrogate series, and reports the Student *t* paired statistics of the comparison between original and surrogate data. The figure shows a coherency modulus significantly greater for the original than for the surrogate data over the whole frequency axis, the difference likely reflecting both the feedback baroreflex SBP-IBI coupling and the feedforward mechanical IBI-SBP coupling.

Surrogate data also allows us defining the minimal threshold C_th_ to be used as one of the two criteria for accepting measures of the SBP-IBI transfer function over the LF band as BRS estimates (the second criterion is a negative phase). For this aim, we considered again the coherency modulus calculated from the 33 original SBP and IBI time series recorded in control rats and from their 33 derived surrogate time series, and we classified each coherency modulus as belonging to the original or surrogate groups if at least one spectral line was greater than C_th_ in the LF band. We found that the choice of a coherency modulus greater than 0.33 assures the best trade-off between sensitivity (97%) and specificity (97%) of the classification, and therefore we set C_th_ = 0.33 as one of the criteria for estimating BRS with the transfer function method.

Methods of circular statistics were used for the descriptive statistics of the SBP-IBI phase^83^. Comparisons between phases at different times from SAD were made by the Mann Whitney U test. Phases are estimated reliably when the coherence is high, and we compared phases only for frequency bands with coherency modulus higher than C_th_. The effect of stress on mean values and log-transformed spectral powers was tested by repeated-measure ANOVA with “Time” (i.e., 1-day SAD, 3-day SAD and 7-month SAD) and “Stress” (i.e., baseline vs. air-jet stimulus) as factors.
